# Enrichment of Anaerobic Syngas-Converting Communities and Isolation of a Novel Carboxydotrophic *Acetobacterium wieringae* Strain JM

**DOI:** 10.3389/fmicb.2020.00058

**Published:** 2020-01-31

**Authors:** Ana L. Arantes, João P. C. Moreira, Martijn Diender, Sofiya N. Parshina, Alfons J. M. Stams, M. Madalena Alves, Joana I. Alves, Diana Z. Sousa

**Affiliations:** ^1^Laboratory of Microbiology, Wageningen University & Research, Wageningen, Netherlands; ^2^Centre of Biological Engineering, University of Minho, Braga, Portugal; ^3^Laboratory of Microbiology of Anthropogenic Habitats of Winogradsky Institute of Microbiology, Federal State Institution (Fundamentals of Biotechnology) of the Russian Academy of Sciences, Moscow, Russia

**Keywords:** carbon monoxide, syngas, carboxydotrophs, acetogens, *Acetobacterium*

## Abstract

Syngas is a substrate for the anaerobic bioproduction of fuels and valuable chemicals. In this study, anaerobic sludge was used for microbial enrichments with synthetic syngas and acetate as main substrates. The objectives of this study were to identify microbial networks (in enrichment cultures) for the conversion of syngas to added-value products, and to isolate robust, non-fastidious carboxydotrophs. Enrichment cultures produced methane and propionate, this last one an unusual product from syngas fermentation. A bacterium closely related to *Acetobacterium wieringae* was identified as most prevalent (87% relative abundance) in the enrichments. *Methanospirillum* sp. and propionate-producing bacteria clustering within the genera *Anaerotignum* and *Pelobacter* were also found. Further on, strain JM, was isolated and was found to be 99% identical (16S rRNA gene) to *A. wieringae* DSM 1911^T^. Digital DNA-DNA hybridization (dDDH) value between the genomes of strain JM and *A. wieringae* was 77.1%, indicating that strain JM is a new strain of *A. wieringae*. Strain JM can grow on carbon monoxide (100% CO, total pressure 170 kPa) without yeast extract or formate, producing mainly acetate. Remarkably, conversion of CO by strain JM showed shorter lag phase than in cultures of *A. wieringae* DSM 1911^T^, and about four times higher amount of CO was consumed in 7 days. Genome analysis suggests that strain JM uses the Wood-Ljungdahl pathway for the conversion of one carbon compounds (CO, formate, CO_2_/H_2_). Genes encoding bifurcational enzyme complexes with similarity to the bifurcational formate dehydrogenase (Fdh) of *Clostridium autoethanogenum* are present, and possibly relate to the higher tolerance to CO of strain JM compared to other *Acetobacterium* species. *A. wieringae* DSM 1911^T^ grew on CO in medium containing 1 mM formate.

## Introduction

In the frame of a circular bio-economy, it is essential to develop technologies for the sustainable conversion of waste materials to fuels and chemicals. Solutions combining the gasification of low-biodegradable wastes, such as lignocellulosic materials, plastic-based wastes, or municipal solid waste, with the biological conversion of the generated syngas have been subject of growing interest and show excellent perspectives (Bengelsdorf et al., 2018; Yasin et al., 2019). Some microbes can grow on carbon monoxide (CO) and/or CO_2_/H_2_, which are the main components in syngas. Acetogenic organisms are used in commercial syngas fermentation, such as the LanzaTech® process, to produce ethanol from CO-rich streams (Dürre and Eikmanns, 2015; Molitor et al., 2016; De Tissera et al., 2017; Redl et al., 2017). Carboxydotrophic acetogens are phylogenetically diverse and have been isolated from a variety of habitats including soil, sediments, intestinal tracts of animals and humans (Diender et al., 2015). Acetogens utilize the Wood-Ljungdahl pathway (WL pathway), also known as reductive acetyl-CoA pathway, to conserve energy for growth and perform CO_2_ fixation (Ragsdale and Pierce, 2008). The most studied acetogenic bacteria include *Acetobacterium woodii, Clostridium ljungdahlii, Clostridium autoethanogenum, Clostridium carboxidivorans, Eubacterium limosum, Moorella thermoacetica*, and *Moorella thermoautotrophica* (Bengelsdorf et al., 2018; Müller, 2019). With C1-compounds, some acetogens mainly produce acetate, while others also produce alcohols, such as butanol and hexanol (Diender et al., 2015; Phillips et al., 2015; Abubackar et al., 2016, 2018; Bengelsdorf et al., 2018).

In this work, anaerobic sludge, previously acclimatized to syngas in a continuous bioreactor (Pereira, 2014), was used to start the enrichment of microorganisms capable of converting CO/syngas. Analysis of microbial communities in enrichment cultures allowed the identification of a predominant acetogen closely related to *Acetobacterium wieringae*, together with bacteria clustering within *Anaerotignum* and *Pelobacter* genera. A novel carboxydotrophic acetogen, *A. wieringae* strain JM, was isolated. Growth of strain JM on CO was compared with that of *A. wieringae* DSM 1911^T^ and *A. woodii* DSM 1030^T^.

## Materials and Methods

### Media and Microorganisms

The basal medium for the cultivation of the microbial cultures contained the following (l^−1^): Na_2_HPO_4_.2H_2_O, 0.53 g; KH_2_PO_4_, 0.41 g; NH_4_Cl, 0.3 g; CaCl_2_.2H_2_0, 0.11 g; MgCl_2_.6H_2_0, 0.10 g; NaCl, 0.3 g; NaHCO_3_, 4.0 g; and Na_2_S. 9H_2_0, 0.48 g [as well as acid and alkaline trace elements (each, 1 ml/liter) and vitamins (0.2 ml/liter) prepared as described by Stams et al. (1993)]. For incubations with 100% CO, phosphate buffer medium was used and prepared as described previously by Alves et al. (2013). The headspace of the bottles was pressurized to 170 kPa with 100% (v/v) CO, syngas mixture [CO, H_2_, and CO_2_ (60:30:10%, v/v)] or H_2_-free syngas [CO, N_2_, and CO_2_ (60:30:10%, v/v)]. The final pH of the media was 7.0–7.2. Medium was autoclaved and before inoculation supplemented with vitamins and reduced with 0.8 mM sodium sulfide (Na_2_S·7-9H_2_O; Stams et al., 1993).

Anaerobic granular sludge from a multi-orifice baffled bioreactor (MOBB) (temperature: 35–37°C; pH: 5.8–6.7) fed with a syngas mixture (60% CO, 30% H_2_, and 10% CO_2_ (v/v); Pereira, 2014) was used as inoculum for enrichment. *Acetobacterium wieringae* (DSM 1911^T^) and *A. woodii* (DSM 1030^T^) were purchased from DSMZ (German Collection of Microorganisms and Cell Culture, Braunschweig, Germany).

### Enrichment Cultures and Isolation of Strain JM

Enrichment cultures were coded as culture JM(x), where x represents the number of successive transfers (in a total of 18 transfers). Enrichments were started by inoculation of anaerobic sludge (5%, v/v) in anaerobic basal medium (described above). First incubations were done with 170 kPa of syngas [CO, H_2_ and CO_2_ (60:30:10%, v/v)]; acetate (20 mM) was added to the medium as a trial to promote solventogenic metabolism and divert acetogenesis; no yeast extract or formate were supplemented. Cultivation of enrichments was done under non-shaking conditions at 37°C and pH 7.0.

Growth of the highly enriched culture JM(16) was tested using a syngas mixture [60% CO, 30% H_2_, and 10% CO_2_ (v/v)] (total pressure 170 kPa) with or without acetate (20 mM). The microbial communities of cultures JM(7) and JM(16) were accessed by 16S rRNA gene analysis (cloning and sequencing, and Illumina® sequencing).

Culture JM(16) was used for the isolation of *Acetobacterium wieringae* strain JM (the most dominant bacterium in that enrichment). Strain JM was further enriched by using dilution technique (up to 10^−10^), using medium described above and supplemented with 1 mM of formate and under a headspace of 60% CO and 40% N_2_ (v/v) (total pressure 170 kPa). The resulting culture was inoculated in roll tubes with 1.5% low melting point agarose (using the same medium and headspace composition) and incubated at 37°C. Colonies were picked and inoculated in fresh liquid phosphate-buffered basal medium supplemented with 1 mM of formate and 0.1 g/l of yeast extract and under a headspace of 60% CO and 40% N_2_ (v/v) (total pressure 170 kPa), and incubated at 37°C statically. Purity was checked by phase contrast microscopy using a Leica DM2000 microscope (Leica, Microsystems, Weltzar, Germany) and by direct sequencing of the 16S rRNA gene (GATC Biotech, Konstanz, Germany).

### Characterization of Strain JM

The optimum and range of temperature for growth, and ability of growth with different soluble (final concentration of 20 mM) and gaseous (total pressure 170 kPa) substrates were tested. Substrates tested included: D-fructose, D-glucose, sucrose, xylose, lactate, formate, glycerol, ethanol, methanol, pyruvate, fumarate, citrate, glycine, malate, mannitol, galactose, melibiose, glutamate, galactitol, sorbitol, lactose, maltose, serine, H_2_/CO_2_ [80:20% (v/v)], CO [100% (v/v)], CO [50% (v/v)], CO [50% (v/v)] plus acetate, and mixture of CO + H_2_/CO_2_ [Syngas: 60% CO, 30% H_2_, and 10% CO_2_ (v/v)]. Substrate tests were done at the optimum temperature (30°C) and shaken at 130 rpm. Additionally, comparison tests of strain JM and type strains *A. wieringae* DSM 1911^T^ and *A. woodii* DSM 1030^T^ were also done at 30°C and at 130 rpm shaking using CO (50%, 170 kPa); medium was supplemented with 20 mM acetate and 1 mM formate. In these experiments CO was refilled as it was consumed.

### DNA Isolation, PCR, Sequencing, and Phylogenetic Analysis

Twenty milliliter of enrichment cultures JM(7) and JM(16) were used for DNA extraction using the FastDNA SPIN kit for soil (MP Biomedicals, Solon, OH), according to the manufacturer's instructions. Bacterial and archaeal 16S rRNA gene fragments were amplified by PCR, using respectively the primer sets 27F/1492R (Nübel et al., 1996) and A109F/1386R (Gagliano et al., 2015). PCR programs and reaction mixtures used were as described elsewhere (Sousa et al., 2007). The PCR products were purified and cloned in *Escherichia coli* XL-blue competent cells (Agilent Technologies, Santa Clara, CA) as previously described by Sousa et al. (2007). Plasmid amplification and sanger sequencing was done by GATC biotech (Konstanz, Germany). For bacterial isolates, colony PCR was performed using the same primer set and programme described above, and PCR products were sent to GATC biotech (Konstanz, Germany) for sequencing. 16S rRNA gene sequences were assembled with DNA baser software version 4.36.0 (Heracle BioSoft S.R.L, http://www.dnabaser.com) and further compared with the GenBank database (Altschul et al., 1990) using the NCBI BLAST search tool. Illumina Miseq platform sequencing was performed at the research and testing laboratory—RTL Genomics (Lubbock, TX). The MiSeq method used was the Illumina two-step using universal primers for bacteria and archaea, 515f and 806r developed by Caporaso et al. (2011). After sequencing, the data were processed using the data analysis pipeline from RTL, which consists in two major steps, the denoising and chimera detection step and the microbial diversity analysis step, as described in the company procedures.

The 16S rRNA gene sequence of strain JM was submitted to the European Nucleotide Database (ENA) and is available under the accession number LR655884. All the other 16S rRNA gene sequences obtained were submitted to ENA, under the following accession numbers: clones sequences (Sanger sequencing)—from LR657299 to LR657303; sequences from Illumina MiSeq platform—project PRJEB33623.

### Genome Sequencing, Assembling, and Annotation

DNA was extracted from 50 mL of a grown culture of strain JM using MasterPure^TM^ Gram positive DNA purification Kit (Epicenter, Madison, WI). DNA quality was checked by electrophoresis in a 0.8% (w/v) agarose gel, using a mass standard (lambda phage DNA) and a size marker (Hind III digested lambda phage DNA). The genome of strain JM was sequenced using Illumina HiSeq X Ten platform (Illumina Inc., San Diego, CA) at Novogene (Beijing, China). Genome was assembled using a pipeline comprising: Ray (Boisvert et al., 2012) to generate an initial assembly, followed by Opera (Gao et al., 2011) for genome scaffolding, and CAP3 (Huang and Madan, 1999) for assembling optimization. For Ray assembler, the optimal kmer size was calculated with KmerGenie (Chikhi and Medvedev, 2014). Automated annotation was performed using the RAST annotation server (Aziz et al., 2008), followed by manual curation. Digital DNA-DNA hybridization value (dDDH) of strain JM and *A. wieringae* DSM 1911^T^ were obtained using the Genome-to-Genome Distance Calculator 2.1 (GGDC; https://ggdc.dsmz.de; Meier-Kolthoff et al., 2013, 2014).

The Whole Genome Shotgun project of *Acetobacterium wieringae* strain JM has been deposited at DDBJ/ENA/GenBank under the accession VSLA00000000.

### Analytical Techniques

Organic acids and alcohols were analyzed via high pressure liquid chromatography (HPLC) equipped with a MetaCarb 67H column (Agilent Technologies, Santa Clara, CA). The column was operated at a temperature of 45°C with a flow rate of 0.8 ml min^−1^. Detection was done via a RI and UV detector. 0.01 N H_2_SO_4_ was used as eluent. Samples of 1.0 ml were taken and immediately centrifuged at 13,000 *g*. Subsequently, vials for HPLC analysis were prepared with the supernatant and 30 mM of arabinose solution with the ratio of 8:2 (v/v). Gas analysis was done by gas chromatography (GC). Gas samples of 0.2 ml were taken using a 1 ml syringe and analyzed in a Compact GC 4.0 (Global Analyser Solutions, Breda, The Netherlands). CO, CH_4_, and H_2_ were measured using a molsieve 5A column operated at 100°C coupled to a Carboxen 1010 pre-column. CO_2_ was measured using a Rt-Q-BOND column operated at 80°C. Detection was done via a thermal conductivity detector.

## Results

### Physiological and Microbial Characterization of Enrichment Culture JM

**Figure 1:**
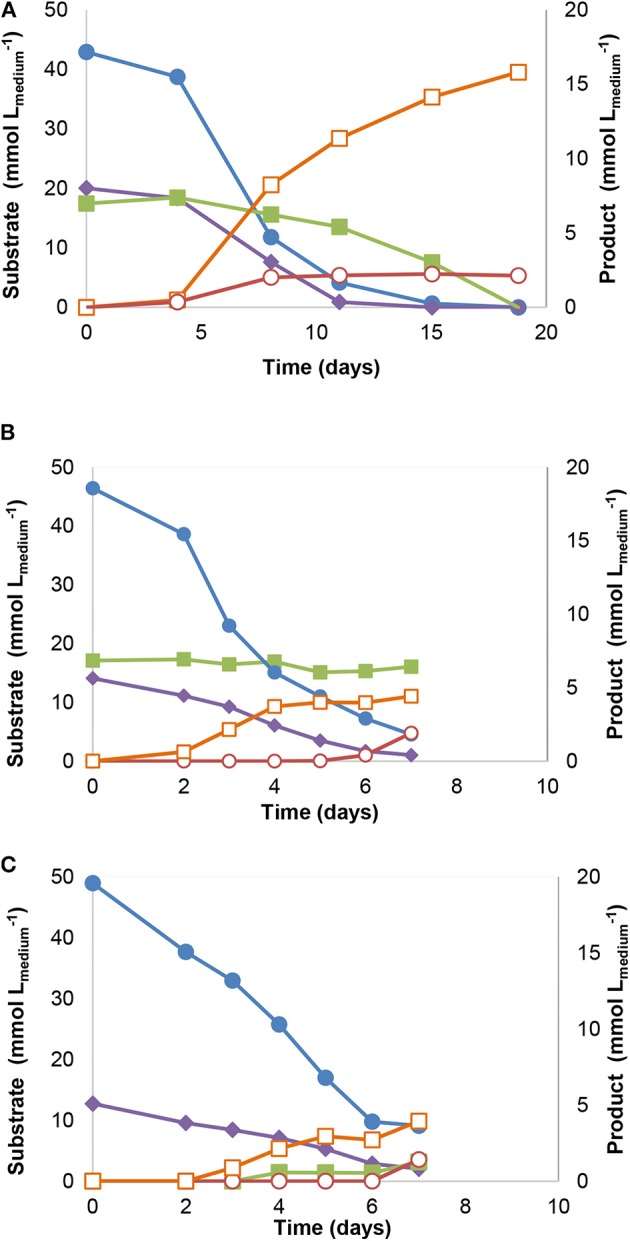
Substrate consumption and product formation by stable enrichment JM cultures with different substrates: **(A)** Syngas and acetate [JM(7)], **(B)** Syngas and acetate [JM(16)] and **(C)** Syngas [JM(16)]. Symbols: (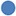) carbon monoxide, (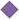) hydrogen, (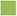) acetate, (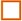) methane, and (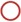) propionate.

Incubation and several transfers of anaerobic sludge with syngas and acetate as substrates, resulted in an enriched culture (culture JM), producing methane and propionate. Substrate consumption and product formation by culture JM(7) are shown in Figure 1: syngas (43 mmol ${\text{L}}_{\text{medium}}^{-1}$ of CO and 20 mmol ${\text{L}}_{\text{medium}}^{-1}$ of H_2_) and acetate (17 mM) were completely converted and resulted in 16 mmol ${\text{L}}_{\text{medium}}^{-1}$ of methane and 2.4 mM of propionate (Figure 1A). In subsequent transfers, acetate consumption by the enrichment cultures stopped as shown for culture JM(16) (Figure 1B). When only syngas was added to the culture as substrate, acetogenic activity could be observed (Figure 1C).

The microbial diversity of the enriched culture JM(7) consisted for about 50% of bacteria affiliated with the genus *Acetobacterium*, while the most abundant methanogen was closely related to *Methanospirilum hungatei* (24%) (Table 1A). In culture JM(16), an organism closely related to *A. wieringae* DSM 1911^T^ (99% of 16S rRNA gene identity) was highly prevalent (87%) (Table 1B). A small fraction of organisms (3%) was related to known propionate producers, namely *Anaerotignum neopropionicum* strain DSM 3847^T^ (former *Clostridium neopropionicum*) (97% of 16S rRNA gene identity) and *Pelobacter propionicus* DSM 2379 ^T^ (92% of 16S rRNA gene identity). From the archaeal domain, *Methanospirillum hungatei* was most dominant (94% of the archaeal clones; Table 1B).

**Table 1:** Microbial community analysis of cultures JM(7) and JM(16).

**(A) Microbial community analysis of culture JM(7) – Illumina MiSeq**				
	**Closest relatives**	**Number (%)** _**(a)**_	**Query Coverage (%)**	**Identity (%)**
Bacteria	*Acetobacterium* sp. (*Acetobacterium* sp. strain SVCO-15 16S ribosomal RNA gene, partial sequence) _(b)_	50	100	99
	*Desulfovibrio* sp. (*Desulfovibrio* sp. S10 gene for 16S ribosomal RNA, partial sequence) _(b)_	8	100	100
Archaea	*Methanospirillum* sp. (*Methanospirillum hungatei* strain JF-1 16S ribosomal RNA gene, complete sequence) _(b)_	24	93	99
**(B) Microbial community analysis of culture JM(16) – Cloning and Sanger Sequencing**				
	**Closest relatives**	**Relative abundance (%)** _**(c)**_	**Query coverage (%)**	**Identity (%)**
Bacteria	*Acetobacterium wieringae* (*Acetobacterium wieringae* strain DP9 16S ribosomal RNA gene, partial sequence) _(d)_	87	98	99
	*Anaerotignum neopropionicum* (*Anaerotignum neopropionicum* strain DSM 3847 16S ribossomal RNA, partial sequence) _(d)_	2	94	97
	*Pelobacter propionicus* (*Pelobacter propionicus* strain DSM 2379 16S ribossomal RNA, partial sequence) _(d)_	1	94	92
Archaea	*Methanospririllum hungatei* (*Methanospirillum hungatei* JF-1, complete genome) _(d)_	94	95	99
	*Methanothrix soehngenii* (*Methanothrix soehngenii* GP6, complete genome) _(d)_	4	94	99

a *Percentage calculated based on the number of sequence counts obtained for the total community by Illumina sequencing, 27817*.

b *Results of sequence alignment by using BLAST toward the NCBI nucleotide database of partial 16S rRNA gene sequences (~291 bp; results obtained from amplicon Illumina sequencing)*.

c *Percentage calculated based on the total number of clones obtained for each domain: 96 clones for Bacteria and 96 clones for Archaea*.

d *Results of sequence alignment by using BLAST toward the NCBI nucleotide database of partial 16S rRNA gene sequences (~1,000 bp; results obtained from cloning and sequencing)*.

### Isolation and Physiological Characterization of *Acetobacterium wieringae* Strain JM

Isolation of strain JM was done by 10-fold dilution series (up to 10^−10^) of culture JM(16), using CO as sole carbon and energy source. After several rounds of dilution series in liquid and solid media, a pure culture (strain JM) was obtained. The 16S rRNA gene sequence was 99% identical to that of *A. wieringae* DSM 1911^T^. Digital DNA-DNA hybridization (dDDH) between strain JM and *A. wieringae* DSM 1911^T^ was 77.1%, which is above the 70% cut-off value generally recommended for species differentiation (Meier-Kolthoff et al., 2013). These results indicate that strain JM is a novel *A. wieringae* strain.

**Figure 2:**
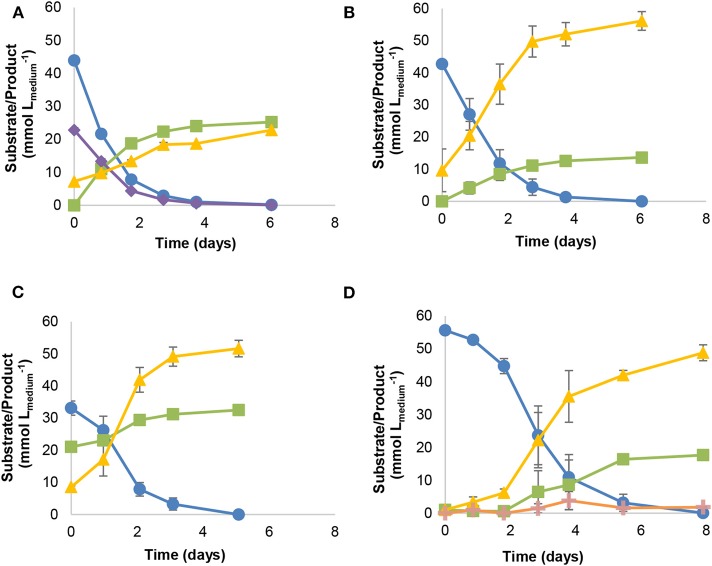
Batch growth of strain JM with different substrate combinations: **(A)** syngas, **(B)** 50% CO, **(C)** 50% CO and acetate (20 mM), **(D)** 100% CO. Symbols: (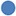) carbon monoxide, (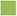) acetate, (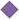) hydrogen, (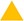) carbon dioxide, (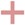) ethanol. All cultures grown using basal medium, without supplementation with yeast extract or formate.

Strain JM is a rod-shaped bacterium with an optimal temperature for growth at 30°C (growth between 20 and 37°C). Strain JM can utilize and grow on CO, without the need of supplementation with yeast extract or formate. Growth on syngas (60% CO, 30% H_2_, and 10% CO_2_, 170 kPa), CO (50% CO and 50% N_2_, 170 kPa), CO (50% CO and 50% N_2_, 170 kPa) plus acetate, and CO (100%, 170 kPa) yielded acetate and CO_2_ (Figure 2). Growth on syngas (Figure 2A) led to the production of higher amounts of acetate (25.3 ± 0.8 mM) and lower CO_2_ accumulation (22.9 ± 0.9 mM) than growth on 50% CO (13.7 ± 0.1 mM acetate, 56.1 ± 2.9 mM CO_2_; Figure 2B). When acetate was added as co-substrate (Figure 2C), lower acetate concentrations were reached (11.5 ± 0.9 mM acetate), though no different fermentation products were detected. On the other hand, growth of strain JM with 100% CO in the headspace (55.6 ± 0.8 mmol ${\text{L}}_{\text{medium}}^{-1}$), yielded ethanol (1.8 ± 0.2 mM) in addition to acetate and CO_2_ (Figure 2D).

The following substrates were tested and utilized by strain JM: H_2_/CO_2_, CO, H_2_/CO_2_ + CO, D-fructose, D-glucose, sucrose, xylose, lactate, formate, glycerol, ethanol, methanol, pyruvate, fumarate, citrate, glycine, malate, mannitol, galactose, melibiose, glutamate, galactitol, and sorbitol. Substrates tested that could not be utilized were: lactose, maltose, and serine.

**Figure 3:**
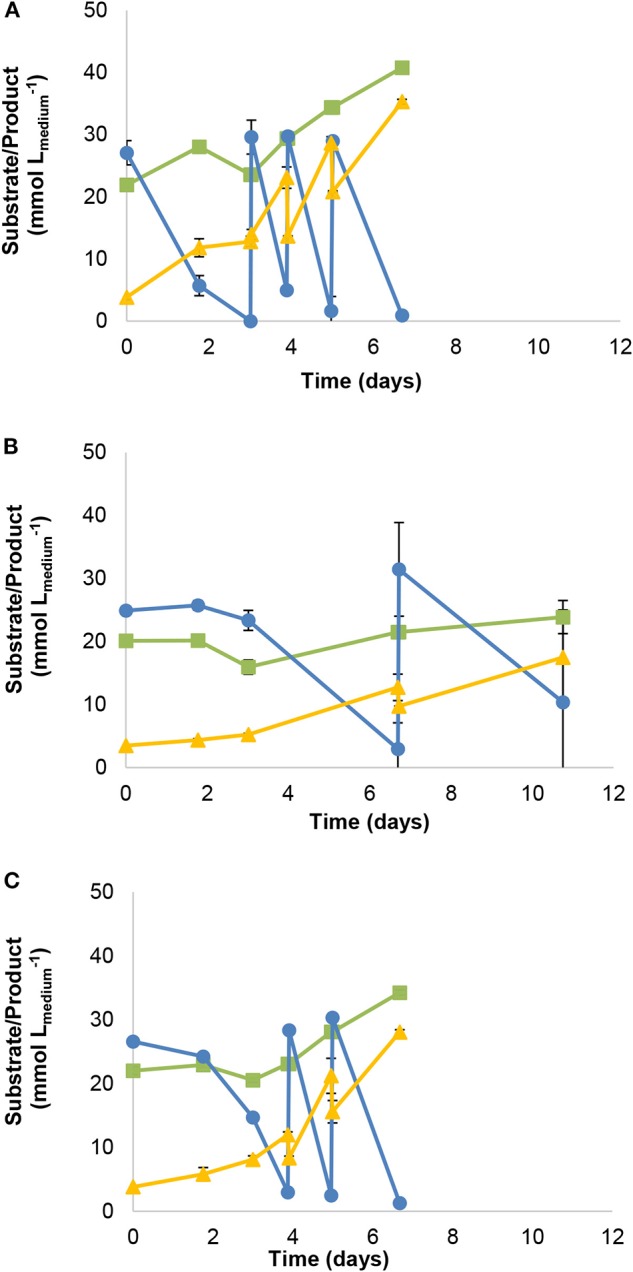
Gas fed-batch growth with CO-acetate of **(A)** strain JM **(B)** *A. wieringae* DSM 1911^T^ and **(C)** *A. woodii* DSM 1030^T^. Symbols: (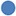) carbon monoxide, (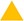) carbon dioxide, (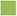) acetate. All cultures grown in basal medium supplemented with 1 mM of formate (without yeast extract).

Parallel growth experiments with CO-acetate as substrates (supplemented with 1 mM formate) were performed for strain JM (Figure 3A), and its closest relatives *A. wieringae* DSM 1911^T^ (Figure 3B) and *A. woodii* DSM 1030^T^ (Figure 3C). CO was refilled to 170 kPa once it was consumed. Strain JM consumed 107.9 mmol ${\text{L}}_{\text{medium}}^{-1}$ of CO in 7 days (Figure 3A). Performance of *A. wieringae* and *A. woodii* during CO conversion was lower: *A. wieringae* consumed 43.1 mmol ${\text{L}}_{\text{medium}}^{-1}$ of CO in 11 days on CO-acetate (Figure 3B), while *A. woodii* was able to convert 78.5 mmol ${\text{L}}_{\text{medium}}^{\text{\_}1}$ of CO in 7 days on CO-acetate (Figure 3C).

### Genome Analysis

**Figure 4:**
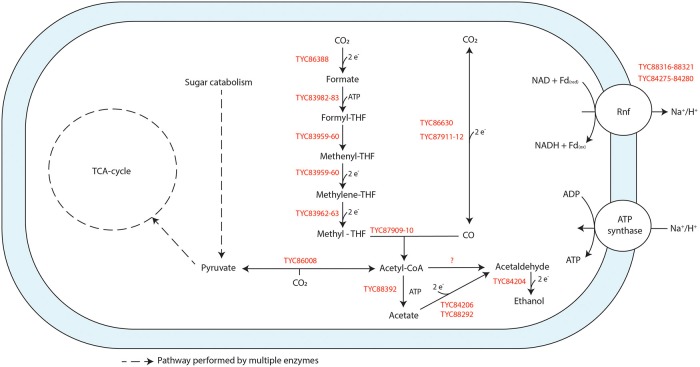
Schematic representation of the physiology of strain JM when grown on CO. Genes found in the genome that are annotated to perform specific reactions are indicated in red. Reactions are not displayed stoichiometrically.

Genome assembly of strain JM produced 44 contigs with an N50 size of 195,031 bp. The draft genome sequence consists of 3.61 Mbp and a G+C content of 44.3 mol%. The genome has 3,240 protein-coding genes, 46 tRNA genes, and 12 rRNA genes. All enzymes of the WL pathway are encoded for in the genome of strain JM (Figure 4), supporting its ability to grow on H_2_/CO_2_ and/or CO. One formate dehydrogenase (Fdh) (TYC86388) was annotated in the genome, showing similarity to the formate dehydrogenase subunit H (FdhH) of the hydrogen-dependent carbon dioxide reductase (HDCR) complex found in *A. woodii* (Bertsch and Müller, 2015). The genes of the HDCR associated hydrogenase were not found in the vicinity of this Fdh. As the Fdh was located at the end of a contig, it is possible that associated hydrogenase subunits were missed. Genes of the rest of the methyl-branch of the WL pathway are located adjacent to each other, including formyl-THF ligase (TYC83982-83), a bifunctional 5,10-methylenetetrahydrofolate dehydrogenase/5,10-methenyltetrahydrofolate cyclohydrolase (TYC83959-60) and a methylene-THF reductase (TYC83962-63). Two carbon monoxide dehydrogenases (*codh*) encoding genes (TYC86630, TYC87911-12) were identified. TYC87911-12 is located in close vicinity to a gene sequence encoding for a acetyl-CoA synthase (*acs*) complex (TYC87909-87910) and thus likely serves a dual function: CO-oxidation and acetyl-CoA formation. TYC 86630 appears to have a CODH catalytic subunit (CooS) motive and is next to an iron-sulfur cluster domain protein, suggesting it encodes for a monofunctional CODH. Several genes in the genome (e.g., TYC85757-59, TYC86583-84) show similarity to bifurcating complexes such as the NADH-dependent reduced ferredoxin:NADP^+^ oxidoreductase (Nfn) complex, or the bifurcating Fdh/[Fe-Fe] hydrogenase complex (Wang et al., 2013). Additionally, two blocks of genes encode for a Ferredoxin:NAD^+^ oxidoreductase (Rnf) complex (TYC 88316-21, TYC84275-84280), typically involved in the build-up of a cation gradient.

Genes encoding for acetate and ethanol formation pathways are present. This includes an acetate kinase (*ack*) (TYC88392) and several alcohol/acetaldehyde dehydrogenase genes (Figure 4). Additionally, the genome contains two acetaldehyde:ferredoxin oxidoreductase genes (TYC88292, TYC84206), of which the latter is located next to a gene coding for an alcohol dehydrogenase. Pyruvate:ferredoxin oxidoreductase (TYC86008) is present for the formation of pyruvate from acetyl-CoA, allowing for assimilation metabolism.

General propionate formation pathways (e.g., methylmalonyl-pathway), are not annotated or not complete in strain JM. Nevertheless, pathways for conversion of propanoyl-CoA to propionate are present, so indirect formation of propionate from e.g., amino acid metabolism is potentially possible.

## Discussion

A novel carboxydotrophic *A. wieringae* (strain JM) was isolated from a syngas-converting enrichment culture, producing mainly acetate and small amounts of ethanol from CO. The fact that *Acetobacterium* species were the most predominant bacteria in the enrichment cultures (Table 1), and acetate one of the main products detected in the enrichments, suggests that this bacterium was the main CO-utilizer in the enrichment cultures. Microorganisms closely related to *A. neopropionicum* (2% of total sequences) and *P. propionicus* (1% of total sequences) were present in the enrichment cultures JM(16) (Table 1), and were likely responsible for propionate production (Figures 1B,C). *A. neopropionicum* and *P. propionicus* are known for their capability to convert ethanol to propionate (Schink et al., 1987; Tholozan et al., 1992; Ueki et al., 2017). These results suggest that a synergistic interaction between *Acetobacterium* species and propionate-forming bacteria was taking place in the enrichments, where *Acetobacterium* is consuming CO to produce acetate and ethanol, and ethanol further used by close relatives to *A. neopropionicum* and *P. propionicus* to form propionate. Such interactions can be relevant for the overall fitness of microbial communities as they influence thermodynamics of the system. Diender et al. (2019) have recently shown a similar synergistic relation in synthetic co-cultures of *Clostridium autoethanogenum* and *Clostridium kluyveri*. In that study, it was shown that the presence of the ethanol-consuming bacterium *C. kluyveri* induced a higher degree of solventogenesis by the carboxydotrophic organism (compared with monocultures of *C. autoethanogenum*). In the present work, we could derive possibly the same type of interaction by natural enrichment of anaerobic sludge, which points out to a possible significance of this process in natural ecosystems too. Methanogens persisted in the enrichments, despite the reported toxicity of CO toward methanogens (Klasson et al., 1991); species closely related to *Methanospirillum hungatei* JF-1 and *Methanothrix soehngenii* GP6 were present in the enriched cultures (Table 1B). There are few methanogens capable of metabolizing CO to methane, belonging to *Methanobrevibacter, Methanosarcina, Methanothermobacter* genera (Diender et al., 2015). However, *Methanospirillum* is only reported to produce methane from H_2_/CO_2_ or formate (Iino et al., 2010), indicating that these microorganisms might be responsible for methane production, using H_2_ and not CO. We previously tested CO utilization by *Methanospirilum hungatei* JF-1 (DSM 864) but no growth was observed (unpublished data).

Strain JM can grow on CO alone (without supplementation of yeast extract, formate or H_2_/CO_2_) (Figure 2). The type strain of *A. wieringae* (DSM 1911) was described by Braun and Gottschalk (1982), but its capability to use CO has not been tested before. Here we show that *A. wieringae* type strain can grow on CO in the presence of formate. The related *A. woodii* can also grow on CO, but only with H_2_/CO_2_ or formate as a co-substrate (Bertsch and Müller, 2015). In *A. woodii*, a hydrogen-dependent carbon dioxide reductase (HDCR) complex has been found responsible for the production of formate from CO_2_, coupling CO_2_ reduction directly to H_2_ oxidation (Bertsch and Müller, 2015). A similar HDCR complex is present in the genome of *A. wieringae* (OFV70223 - OFV70228). Fe-Fe hydrogenases in the HDCR complex were thought to be sensitive to high CO concentrations (Bertsch and Müller, 2015), which could explain the need for formate when *A. woodii* was grown on CO. However, later it was shown that CO inhibition of the HDCR is fully reversible (Ceccaldi et al., 2017). Also, the thermophilic *Thermoanaerobacter kivui* employs a similar HDCR complex and, after prolonged adaptation to CO, was able to grow on 100% CO without formate (Weghoff and Müller, 2016). This suggests that hydrogenases in HDCR complex can adapt to CO. Strain JM was isolated from a long-term enrichment growing on syngas, and this could have resulted in a better adaptation to CO. The genome of strain JM encodes for a formate dehydrogenase with high similarity to the HDCR of *A. woodii*, but the associated hydrogenases were not found. The *fdh* gene of strain JM was located at the end of a contig, and therefore we cannot exclude the possibility of missing part of the sequence of the HDCR. It thus remains unclear if strain JM employs a HDCR, but formate formation does not seem to be a limiting step in its metabolism. Besides adaptation of hydrogenases to CO, a link between the abundance of the monofunctional CODH CooS and the bifunctional CODH/ACS and the efficiency in CO utilization was proposed (Weghoff and Müller, 2016). The genome of strain JM encodes for both, a bifunctional CODH/ACS complex (TYC87911-TYC87912), and an apparent monofunctional CODH (TYC86630). However, as the genomes of both *A. woodii* and *A. wieringae* also appear to carry genes of mono- as well as bi-functional CODH this appears not to make a difference here.

Comparison of CO conversion by strain JM and by the type strains of *A. wieringae* and *A. woodii* shows that strain JM can convert a higher amount of CO during the 7 days of incubation (up to 4- and 2.5-fold higher, respectively) (Figure 3), which again could result from metabolic adaptation to CO during enrichment and isolation of strain JM. Adaptation to CO has been previously shown to play a role to increase the growth rate of *A. woodii* up to 3 fold compared to non-adapted cultures (with maximum 75% CO and 100 mM formate) (Bertsch and Müller, 2015). Duplication times in mesophilic carboxydotrophs range from 4 to 14 hours, with the lowest being achieved by *Clostridium* species (namely *C. ljungdahlii, C. autoethanogenum* and *C. ragsdalei*); *A. woodii* has reported (or calculated) duplication times between 5.5 and 13 hours (Diender et al., 2015).

Strain JM encodes for both the carbonyl and methyl branches of the WL pathway as found in *A. woodii* (Sharak Genthner and Bryant, 1987; Poehlein et al., 2016). Additionally, the genome analysis of *A. wieringae* strain JM revealed the presence of two CODH encoding sequences, explaining its CO utilizing properties. Strain JM could grow in the presence of different initial partial pressures of CO or CO/H_2_, producing acetate and CO_2_; to note, though, that incubation of strain JM with 100% CO (170 kPa) led to the production of ethanol as well (Figure 2D). Both, aldehyde:ferredoxin oxidoreductase (*aor*) (TYC84206, TYC88292) and alcohol dehydrogenase (*adh*) (TYC84204) encoding genes are present in the genome of strain JM, and are potentially linked to ethanol production by this strain. Conversion of carboxylic acids to alcohols via the AOR-ADH pathway has been previously observed in several carboxydotrophs (Simon et al., 1987; Perez et al., 2013), and further genetic evidence for the pathway reported by Basen et al. (2014). The presence of the AOR may contribute for the efficient growth of strain JM on CO, as redox equivalents can be shuttled into ethanol, without interfering with energy conservation (Köpke et al., 2010). Earlier reports on *A. wieringae* type strain show ethanol formation from fructose and H_2_/CO_2_ conversion (Buschhorn et al., 1989; Groher and Weuster-Botz, 2016) and on *A. woodii* from glucose fermentation (Buschhorn et al., 1989), though not with CO. The same authors also reported that *A. woodii* and *A. wieringae* could use ethanol as substrate as well (Buschhorn et al., 1989), which is also the case for strain JM. This can also explain ethanol consumption by strain JM in later phase of CO fermentation (Figure 2D).

## Conclusions

Enrichment cultures mainly composed of strain JM and close relatives to *A. neopropionicum* and *P. propionicus* were able to produce propionate from syngas, which is an uncommon product from syngas fermentation. A novel carboxydotrophic *A. wieringae* strain JM was isolated from the syngas enriched culture. Strain JM could efficiently convert CO to acetate (and CO_2_) and small amounts of ethanol. This is the first report of an *A. wieringae* strain able to use CO, and proof that type strain (DSM 1911^T^) can also utilize CO, but only in the presence of formate. It is also the first report of isolation of an *Acetobacterium* species from a CO-fed enrichment.

## Data Availability Statement

The datasets generated for this study can be found in the 16S rRNA gene sequences submitted to the European Nucleotide Database (ENA) accession numbers LR655884, LR657299 to LR657303, PRJEB33623. The Whole Genome Shotgun project of *Acetobacterium wieringae* strain JM has been deposited at DDBJ/ENA/GenBank under the accession VSLA00000000.

## Author Contributions

DS, AS, and MA proposed and designed the study. DS and JA provided guidance to AA and JM and streamlined communication between the two labs. Research was performed by AA (initial enrichments), JM (characterization of enrichments and isolation of strain JM), SP and AA (physiological characterization of strain JM), and MD (genomic analysis of strain JM). AA drafted the manuscript with support of JA and DS, and revisions by all the authors.

### Conflict of Interest

The authors declare that the research was conducted in the absence of any commercial or financial relationships that could be construed as a potential conflict of interest.
